# Land use impacts on soil aggregate associated organic carbon in reclaimed coal mining subsidence areas: mechanisms and implications

**DOI:** 10.1038/s41598-025-08409-0

**Published:** 2025-07-01

**Authors:** Junfeng Qu, Nan Jiang, Yuting Dai, Ying Yang, Yang Ning, Kun Wang, Fuyao Chen, Jiu Huang, Min Tan

**Affiliations:** 1 School of Public Policy and Management, China University of Mining and Technology, Xuzhou, 221116 Jiangsu China; 2 Carbon Neutral Research Institute, China University of Mining and Technology, Xuzhou, 221116 Jiangsu China; 3 School of Environment and Surveying and Mapping, China University of Mining and Technology, Xuzhou, 221116 Jiangsu China; 4 Xuzhou Institute of Ecological Civilization Construction, Xuzhou, 221116 Jiangsu China; 5 Xuzhou Bioengineering Vocational and Technical College, Xuzhou, 221116 Jiangsu China

**Keywords:** Reclaimed soil, Soil aggregates, Soil organic carbon, Coal mining subsidence area, Land-use patterns, Environmental sciences, Environmental impact

## Abstract

Under the background of ecological restoration and soil structure improvement in coal mining subsidence areas, understanding the impact of land use on soil organic carbon pools and aggregate characteristics is of great significance to evaluate the quality change and ecological effect of reclaimed soil and guide the improvement of reclaimed soil. Although the dynamics of soil organic carbon under land use changes have been studied, the mechanism of aggregate carbon protection in reclaimed mining soil is still unclear. Taking the reclaimed soils of farmland, woodland, and wasteland in the Dongtan mining area of Zoucheng City in Shandong Province as the research object, by comparing the particle size composition of soil aggregates, the distribution characteristics of organic carbon in 0–20 cm and 20–40 cm soil layers, and analyzing the differences of different organic carbon components, it was found that the organic carbon content of aggregates in the 0–20 cm soil layer was higher than that in the 20–40 cm soil layer; In topsoil, the contribution of fine aggregates to total organic carbon was the largest; the contents of free light fraction (free LF) organic carbon in fine aggregates, micro-aggregates, and total free light fraction organic carbon in forest soil were significantly higher than those in farmland and wasteland. These results suggest that fine aggregates may serve as primary carriers of reclaimed soil organic carbon. The study on the surface soil aggregate organic carbon helps reveal the transformation and accumulation process of reclaimed soil organic carbon. The study found that woodland reclamation may enhance microbial-driven soil organic carbon storage, suggesting that targeted land-use strategies have the potential to maximize the carbon sequestration capacity in degraded ecosystems. Findings are specific to the 7-year reclamation period and Dongtan mining area, requiring long-term regional validation.

## Introduction

Globally, underground coal mining provides crucial resources for the process of industrialization and economic development. However, it has also triggered a series of severe ecological and social problems^1^. A great deal of research indicates that mining activities inevitably disrupt the regional ecological balance, and the issue of land subsidence is particularly prominent^2^. China’s coal mining is dominated by underground mining, which accounts for approximately 95% of the raw coal output. The subsidence areas are mainly concentrated in the central and eastern plains, where coal and grain are highly compounded. The land subsidence caused by underground mining has become increasingly evident, continuously disturbing ecological environment factors such as soil and vegetation. In regions with high groundwater levels, the subsidence due to mining always leads to large tracts of land being submerged^3^, making land resources even scarcer. As a result, land reclamation and ecological restoration have become a global focus^4–7^. In addition, the massive damage to the soil threatens national food security and profoundly affects the regional carbon cycle. Land reclamation and ecological reconstruction in the subsidence areas of coal mining with high phreatic levels are urgently needed tasks, and the modification of soil quality and ecological effects is the key to the success of reclamation.

Soil is the largest terrestrial organic carbon pool, and small changes in soil organic carbon (SOC) stocks will have a significant impact on the global carbon cycle, land productivity, and food security^8^. Soil aggregates, as components of soil structure^9^, control the dynamics of SOM and nutrient cycling^10^. The physicochemical protection provided by aggregate structure and mineral surfaces is essential for building and maintaining soil carbon and nitrogen stocks^11^. Compared with natural soil, both the coal mining process and reclamation projects greatly disturb the soil, and the reclaimed soil structure is seriously damaged. Soil organic carbon plays a key role in the early soil formation process and ecological function reconstruction of the reclaimed soil in mining areas^12,13^. During the process of post-reclamation, the soil structure is continuously improved, and organic carbon continuously increases, exhibiting a huge carbon sequestration potential. It has been shown that soil aggregates play a key role in the retention of soil organic carbon (SOC)^14,15^ and that there is a strong interplay between soil organic carbon and aggregates: Soil organic carbon fixation first enhances the stability of aggregates^16,17^, and stable aggregates can provide physical protection for soil organic carbon. Aggregate stability and organic carbon are key indicators in the characterization of soil quality and soil ecological characteristics^18,19^. The distribution of organic carbon in aggregates of different particle sizes is critical to the study of the evolution and sequestration characteristics of organic carbon^20^.

The land-use pattern is one of the factors that affect the soil organic carbon pool, aggregate distribution, and stability characteristics^21^. It has a direct and profound impact on soil development and determines the ability of soil to fix and store organic carbon. Different land-use patterns change the material cycle of the soil and have a greater influence on the changes in the carbon cycle and storage of the regional ecosystem. A large number of studies have been conducted internationally on the impact of different land-use patterns on soil aggregates and organic carbon. Studies have shown that the SOC dynamics vary greatly in terms of land-use changes^22^. Some researchers have elaborated on the differences between reclaimed soil aggregates and organic carbon under different land-use patterns, generally proving that different land-use patterns exert extensive effects on the characteristics of reclaimed soil aggregates and organic carbon^23^, and also affect soil quality and carbon sequestration ability^24,25^. To further reveal the impact of land use on the soil’s organic carbon pool, the researchers selected organic carbon component indicators that were more sensitive than the total organic carbon response to investigate the impact of different land-use patterns on aggregate stability and organic carbon^26,27^. However, most of the existing studies on reclaimed soils have focused on exploring the spatial characteristics of certain soil properties, such as soil nutrients, soil structure, and soil heavy metals^28^. These studies have adequately analyzed the spatial distribution of tacitly specific aspects of reclaimed soils and revealed general trends in reclaimed soils. However, most of the studies have not deeply explored the response mechanisms of specific SOC components (e.g., organic carbon, which is closely associated with different aggregates) under land use changes. Owing to the same similarities of the measured organic carbon components and the lack of systematic indicators, however, the results of the soil organic carbon components were quite different. The organic carbon differences under different land-use patterns are affected by the soil’s stabilization mechanism, which is mainly related to the various protective abilities of aggregates with different particle sizes and different turnover times^29–31^. Therefore, researchers generally separate the organic carbon components closely related to agglomerates by combining density and size and studying the combination and stability characteristics of different components^32,33^.

The reclaimed soil has its unique laws of evolution and development under the new soil-forming conditions. Investigating the soil development under different land-use patterns after the reclamation of subsided land will help the ecological restoration of mining areas and the improvement of soil structure. In this study, we will focus on specific SOC components, analyze the distribution characteristics of organic carbon in various particle-level aggregates under different land-use practices, and explore the intrinsic connection between land-use practices and microbial- and plant-driven carbon pathways, to clarify the influence of reclaimed soil on the physical protection mechanism of soil organic carbon under different utilization modes, to provide a scientific basis for the rational use of land after reclamation.

## Materials and methods

### Study site

**Fig. 1:**
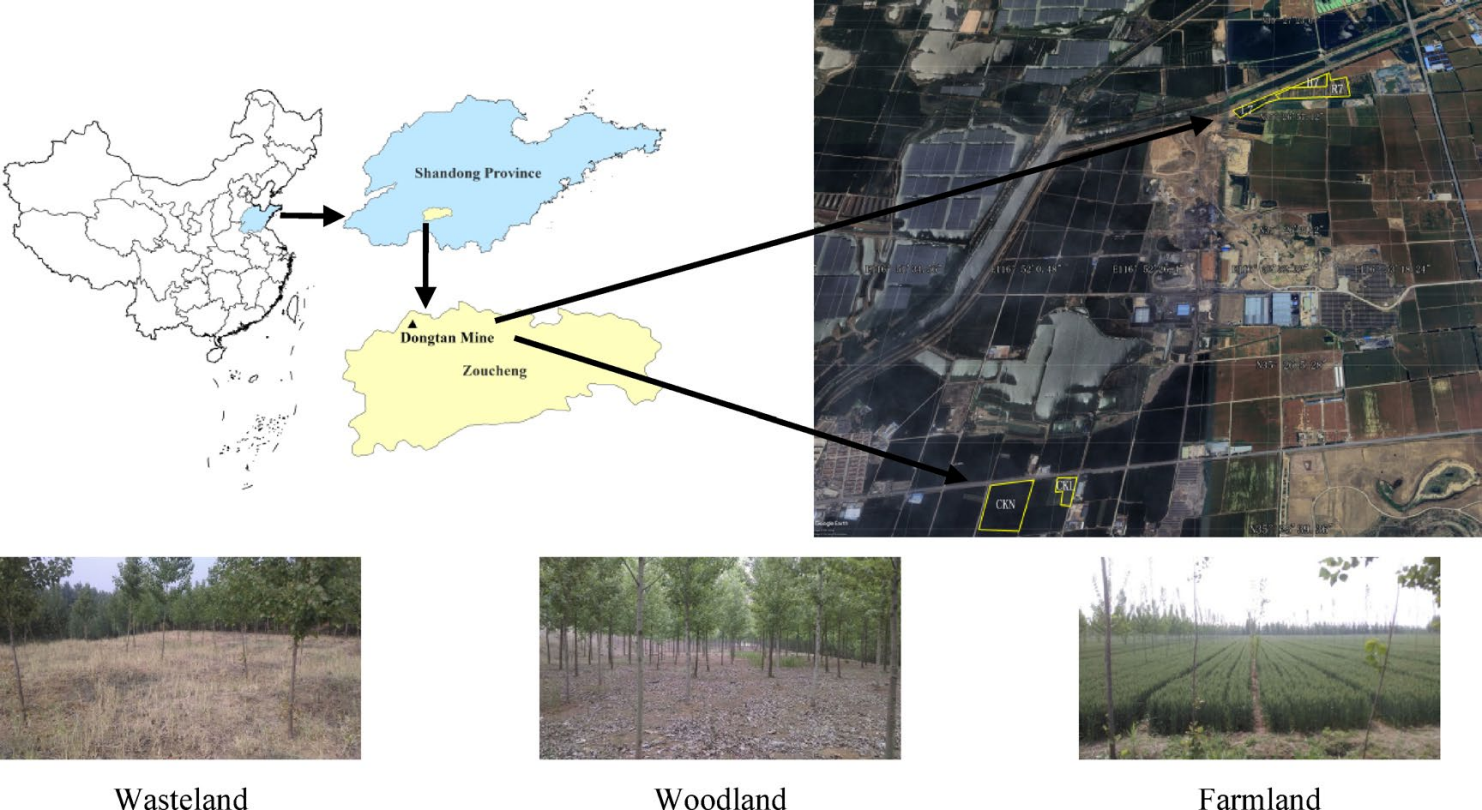
Geographic locations of the experimental sites. (Software: ArcGIS 10.2, https://www.esri.com/).

The study area was located in the Dongtan mining region (Fig. 1) in Zoucheng City, Shandong Province, China (35°8′12″–35°32′54″N, 116°46′30″–117°28′54″E). This area has a warm-temperate transitional monsoon climate, with four distinct seasons and simultaneous rain and heat. The Dongtan Coal Mine is situated in an area of China with a high phreatic water level and began operation in 1989. The underground phreatic water level is generally − 1.5 m. Long-term coal mining has caused serious surface subsidence. At present, stagnant water areas with depths of approximately 4–10 m have formed in many places. The coal gangue reclamation site project in the coal mining subsidence area was implemented from 2001 to 2011. The reclamation process consisted of coal gangue-filling reclamation. The specific process comprised the following. First, the topsoil was pre-stripped from the surface of the mining areas with no early-stage subsidence and was stored for later use. Second, after the coal mining subsidence areas were stabilized, the water in the subsidence basin was removed, and the debris in the basin was cleaned. In addition, the compaction parameters and the amount of coal gangue were determined through preliminary experiments. Based on the results of the preliminary experiments, the subsidence basin was backfilled with coal gangue to the design elevation (averaging 47.0 m) using leveling and compacting, with the thickness of the gangue filling layer ranging from approximately 2–4 m. Finally, the pre-stripped and stored topsoil was backfilled and leveled. The thickness of the topsoil covering was about 0.6–1.0 m, and crops or other vegetation were subsequently planted on the reclaimed site after leveling.

### Collection of mine soil samples

The experimental plots in this study were located in the reclamation area of the Dongtan Coal Mine in Zoucheng City. The sample plots were reclaimed in 2009 with different land-use patterns, including farmland (R7), woodland (L7), and wasteland (H7). The surrounding normal farmland (CKN) and normal woodland (CKL) that had not undergone reclamation served as controls (Table 1). We collected samples in April 2016. A total of five sample plots were set up based on the land-use patterns, and three sampling points were positioned in each sample plot. For each sample point, a stainless-steel sampler with an inner diameter of 3.5 cm and a length of 50 cm was employed to extract six tubes of soil at depths of 0–20 cm and 20–40 cm, and then mix them into a soil sample by layer. An attempt was made to avoid squeezing the collected soil samples, and they were then brought back to the laboratory and dried at room temperature. When the soil moisture content reached the plastic limit of the soil, small soil blocks were formed with a diameter of < 1 cm along the natural cracks of the soil. Debris was removed, and the soil was passed through an 8-mm sieve and allowed to air dry. The air-dried soil samples were then used for soil aggregate grouping and organic carbon determination.

**Table 1:** Basic characteristics of sample plots.

Site	Year of reclamation	Years since reclamation	Land-use pattern	Soil	Plants
R7	2009	7	Farmland	Fluvo-aquic soil	Soybean-wheat rotation
L7	2009	7	Woodland	Fluvo-aquic soil	Poplar
H7	2009	7	Wasteland	Fluvo-aquic soil	Weeds
CKN	—	—	Farmland	Fluvo-aquic soil	Soybean-wheat rotation
CKL	—	—	Woodland	Fluvo-aquic soil	Poplar

### Experimental method

**Fig. 2:**
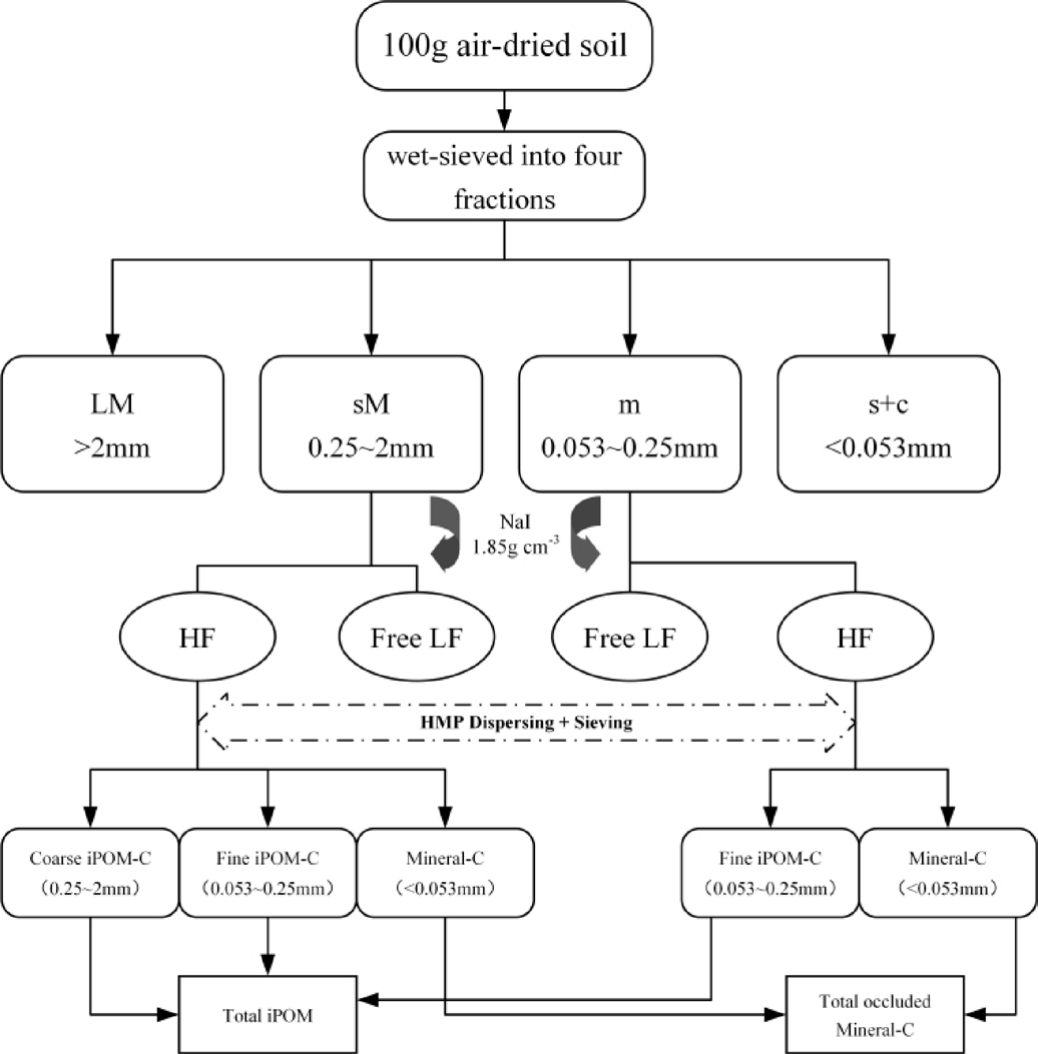
Separation steps of the aggregate-associated organic carbon fraction (Software: Microsoft Visio 2021 https://www.microsoft.com/en-us/microsoft-365/visio/flowchart-software). LM: large macroaggregates; sM: small macroaggregates; m: microaggregates; s + c: silt-clay aggregates; HF: Heavy fraction; Free LF: free light fraction; Coarse iPOM-C: Coarse intra - aggregate particulate organic carbon; Fine iPOM-C: Fine intra - aggregate particulate organic carbon; Mineral-C: Mineral - bound carbon; HMP: Sodium hexametaphosphate.

In this study, the wet sieve method^34^ was employed to group the particle sizes of the soil aggregates. The organic carbon component of the soil aggregates was separated using the density-combination method developed by Six et al.^35^, which integrates particle size fractionation and density centrifugation to characterize physically protected carbon pools. For details, see the separation flowchart in Fig. 2. Specifically, air-dried soil samples (< 8 mm) were first wet-sieved into four fractions: >2 mm (large macroaggregates, LM), 0.25–2 mm (small macroaggregates, sM), 0.053–0.25 mm (microaggregates, m), and < 0.053 mm (silt-clay aggregates, s + c). For each fraction, 10 g of aggregates were mixed with 35 mL of sodium polytungstate solution (density = 1.85 g/cm³), vacuum-treated for 10 min to remove entrapped air, and centrifuged at 1,250 rpm for 60 min to separate free light fraction (free LF, density < 1.7 g/cm³)—primarily comprising un-decomposed plant residues—from the heavy fraction (density ≥ 1.7 g/cm³)^35^. The heavy fraction was further dispersed with 10 mL of 0.5% sodium hexametaphosphate (HMP) solution via overnight shaking (18 h) and sieved through 0.25 mm and 0.053 mm meshes to isolate intra-aggregate particulate organic carbon (iPOM), which includes coarse (> 0.053 mm) and fine (< 0.053 mm) fractions. Mineral-bound carbon (Mineral-C) was calculated by subtracting iPOM content from the total heavy fraction of carbon, representing organic carbon tightly associated with mineral surfaces.

The potassium dichromate-external heating method was utilized for the determination of organic carbon in the aggregates and their components. The mineral-bound carbon was determined by subtraction. Concerning related research results^36^, this study chose the aggregates with sizes > 0.25 mm (R_0.25_), the mean weight diameter (MWD), the geometric mean diameter (GMD), and the fractal dimension (D) to describe the distribution and stability features of soil water-stable aggregates. The calculation equations are as follows:

1 $${\text{R}}_{{0.{\text{25}}}} = M_{r>0.25}/MT$$

2 $$MWD = \frac{{\sum\nolimits_{{i = 1}}^{n} {(\bar{x}iwi)} }}{{\sum\nolimits_{{i = 1}}^{n} {wi} }}$$

3 $$GMD = \exp \left[ {\frac{{\sum\nolimits_{{i = 1}}^{n} {wi\ln \bar{x}i} }}{{\sum\nolimits_{{i = 1}}^{n} {wi} }}} \right]$$

4 $$D = 3 - \frac{{\log (M_{i} /M_{T} )}}{{\log (\bar{x}i/x\max )}}$$

where $\bar{x}_{i}$ is the average diameter of a certain size aggregate; *w*_*i*_ is the mass fraction of a certain particle size aggregate, *M*_*r*__>0.25_ is the mass of aggregates with a particle size >0.25 mm, *M*_*i*_ is the mass of aggregates with a particle size$<\bar{x}_{i}$, *M*_*T*_ is the total mass of the aggregates, and *x*_*max*_ is the maximum particle size of the aggregates.

The calculation formula for the contribution rate (*Y*) of soil aggregate organic carbon of a certain particle size to total organic carbon is as follows:

5 $$Y = Ci \times Mi/Cw$$

where *C*_*i*_ is the organic carbon concentration of a certain size aggregate (g kg^−^^1^ aggregate), *M*_*i*_ is the mass fraction of a certain size aggregate (%), and *C*_*w*_ is the total soil organic carbon content (g kg^−^^1^).

### Statistical analysis

After the test data were collated using Microsoft Excel 2016, one-way analysis of variance (one-way ANOVA) and Duncan’s multiple range test were performed with SPSS 21.0 software (SPSS Inc., Chicago, IL, USA) to analyze the variance and significance of differences (α = 0.05) under various land-use patterns. SigmaPlot 14.0 (Systat Software, Inc., San Jose, CA, USA) was utilized for drawing.

## Results

### Distribution and stability characteristics of reclaimed soil aggregates under different land-use patterns

#### Distribution characteristics of soil aggregates

**Fig. 3:**
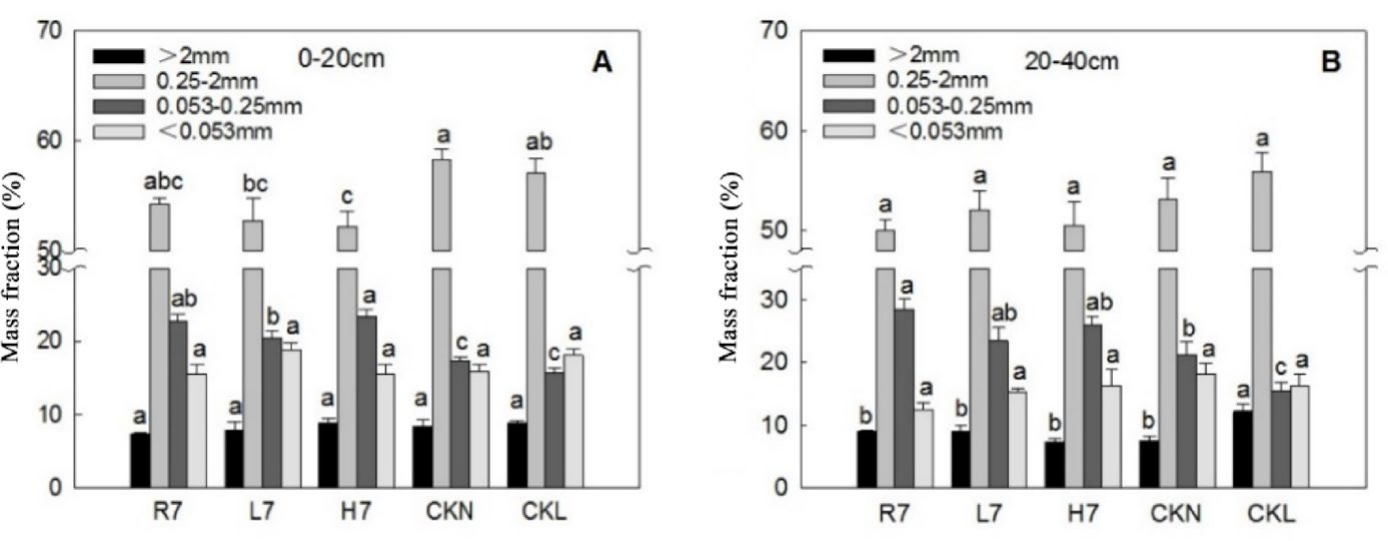
Mass proportion characteristics of soil aggregates under different land-use patterns. Error bars represent standard error (SE); lowercase letters indicate significant differences of the same soil layer among different land-use patterns at *p* < 0.05 according to Duncan’s multiple range tests.

In the 0–20 cm soil layer, the mass fractions of soil aggregates were classified as small macroaggregates (sM; 0.25–2 mm), microaggregates (m; 0.053–0.25 mm), silt-clay aggregates (s + c; < 0.053 mm), and large macroaggregates (LM; > 2 mm), ANOVA showed no significant differences in mass fractions among farmland, woodland, and wasteland (*p* > 0.05, Fig. 3A). Compared to normal farmland and woodland, the sM mass fraction in wasteland was lower, while the microaggregate mass fraction was higher. However, there were no significant differences in the other two types of aggregates.

It can be seen from Fig. 3B that after seven years of reclamation, the relative order of the mass fractions of aggregates of different sizes in the 20–40 cm soil layer of farmland, woodland, and wasteland soil was consistent with that of the 0–20 cm soil layer, and there was also no significant difference in the mass fractions of the four aggregates. Compared with normal farmland, the farmland soil after seven years of reclamation was not significantly different, except for the significantly higher mass fraction of macro aggregates. Farmland had the highest sM mass fraction (54.26%), exceeding woodland (52.75%) and wasteland (52.18%) (Fig. 3A). There was no significant difference among the other aggregate particle sizes.

#### Stability characteristics of soil aggregates

**Fig. 4:**
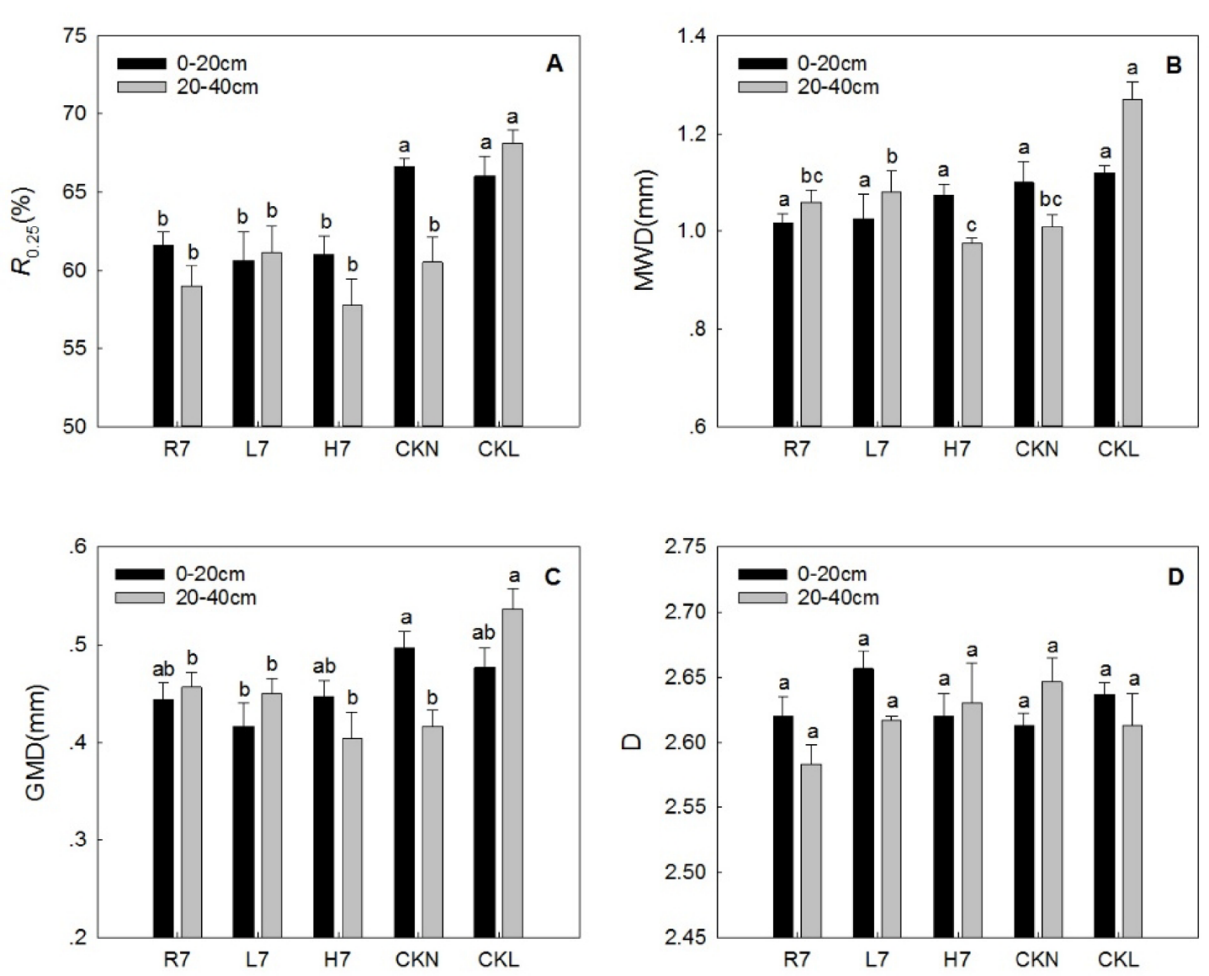
R_0.25_ (**A**), MWD (**B**), GMD (**C**), and D (**D**) characteristics under different land-use patterns. Error bars represent standard error (SE); lowercase letters indicate significant differences of the same soil layer among different land-use patterns at *p* < 0.05 according to Duncan’s multiple range tests.

The different characteristics of the reclaimed soil aggregates, R0.25, MWD, GMD, and D under different land-use patterns are presented in Fig. 4. The R0.25 values in the 0–20 cm soil layer followed CKN = CKL > R7 = H7 = L7 (Fig. 4A), with no significant differences among reclaimed land uses (R7, L7, H7), but all were lower than control sites (CKN, CKL). Specifically, farmland had an R0.25 proportion of 61.63%, woodland 60.65%, and wasteland 61.04% after the seven-year reclamation period. There was no obvious difference among them, but they were all significantly smaller than normal farmland and woodland. Among the different soil layers, the R0.25 of the reclaimed farmland and the normal farmland was greater than the 20–40 cm soil layer in the surface layer, while the R0.25 of the reclaimed woodland and the normal woodland was just the opposite. The R0.25 in the two soil layers of the reclaimed wasteland was smaller than that of the reclaimed farmland and woodland in the corresponding soil layers.

There was no significant difference in the MWD of surface soil aggregates (0–20 cm) under different land-use patterns, while in the 20–40 cm soil layer, the MWD of wasteland was slightly lower than that of reclaimed farmland and woodland (though not statistically significant, Fig. 4B). In the 0–20 cm soil layer, GMD values were CKN > CKL (*p* < 0.05), while H7, R7, and L7 showed no significant differences among themselves (Fig. 4C). Except for the seven-year woodland GMD (0.42 mm), which was significantly smaller than control farmland (CKN, *p* < 0.05), no significant differences were observed among the rest of the sample sites. The GMD characteristics in the 20–40 cm soil layer were consistent with R0.25 results (Fig. 4C), and the fractal dimension D showed no significant differences across land-use patterns (Fig. 4D).

### Organic carbon content and contribution rates of reclaimed soil aggregates under different land-use patterns

#### Organic carbon content of soil aggregates

**Fig. 5:**
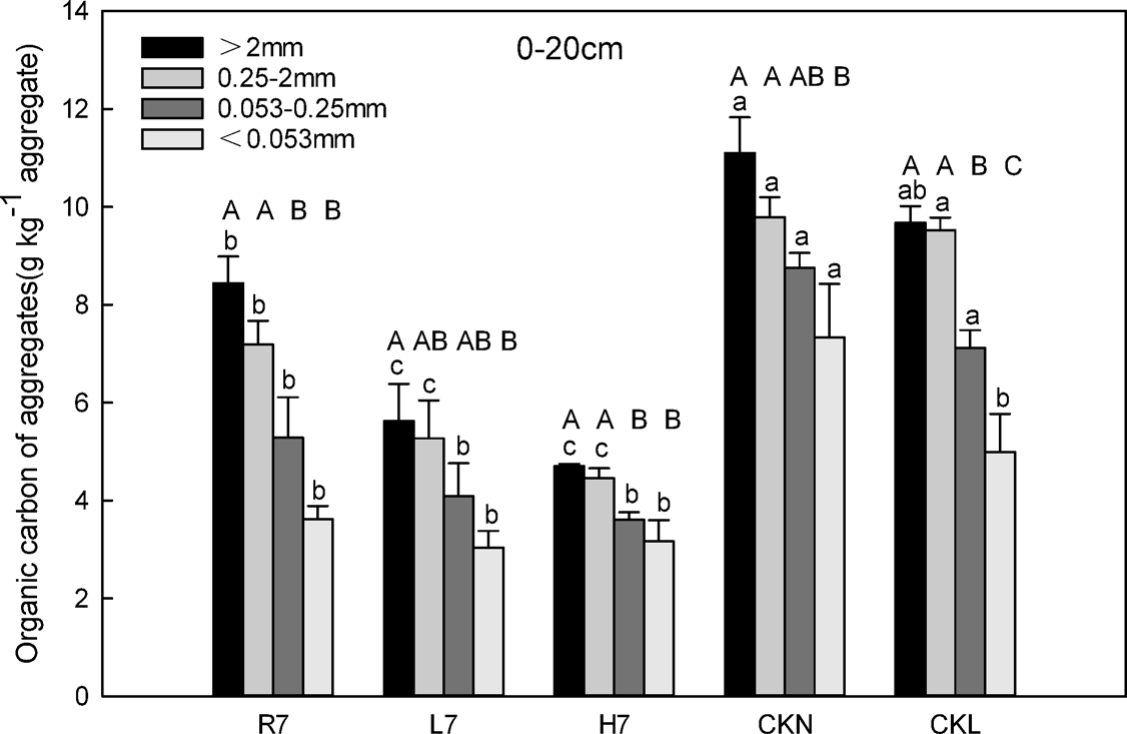
Soil aggregate-associated organic carbon characteristics (0–20 cm) under different land-use patterns. Error bars represent standard error (SE); lowercase letters indicate significant differences of the same particle size among different land-use patterns at *p* < 0.05 according to Duncan’s multiple range tests; capital letters indicate significant differences of the same land-use patterns among different particle sizes at *p* < 0.05 according to Duncan’s multiple range tests.

Land use affects the organic carbon content of soil aggregates. In the 0–20 cm soil layer, the organic carbon content of each particle size in farmland soil after seven years of reclamation was the highest, followed by the content of woodland, while the lowest was a wasteland, which was lower than normal farmland and woodland (*p* < 0.05 Fig. 5). Specifically, the relative order of the content was CKN > CKL > R7 > L7 > H7 (*p* < 0.05). The differences in the organic carbon content of aggregates of various sizes also differed among the three land-use patterns of reclaimed soil. The organic carbon contents of reclaimed farmland sM and m (8.44 g kg^−1^ aggregate and 7.19 g kg^−1^ aggregate, respectively) were somewhat higher than those of woodland and wasteland, while the difference values of woodland (5.63 g kg^−1^ aggregate and 5.27 g kg^−1^ aggregate) and wasteland (4.70 g kg^−1^ aggregate and 4.46 g kg^−1^ aggregate) were small. There were no obvious differences in the organic carbon content of microaggregates and silt-clay aggregates among farmland, woodland, and wasteland.

The particle size distribution of the soil aggregate organic carbon content revealed that the relative order of the organic carbon content of the agglomerates under each land-use pattern was large macroaggregates > small macroaggregates > microaggregates > silt-clay aggregates. Specifically, large macroaggregates in reclaimed farmland, woodland, wasteland, and their respective control sites (normal farmland and woodland) contained significantly higher organic carbon than microaggregates. By contrast, no notable differences were observed in organic carbon content between large macroaggregates and other particle sizes (e.g., small macroaggregates and silt-clay aggregates).

**Fig. 6:**
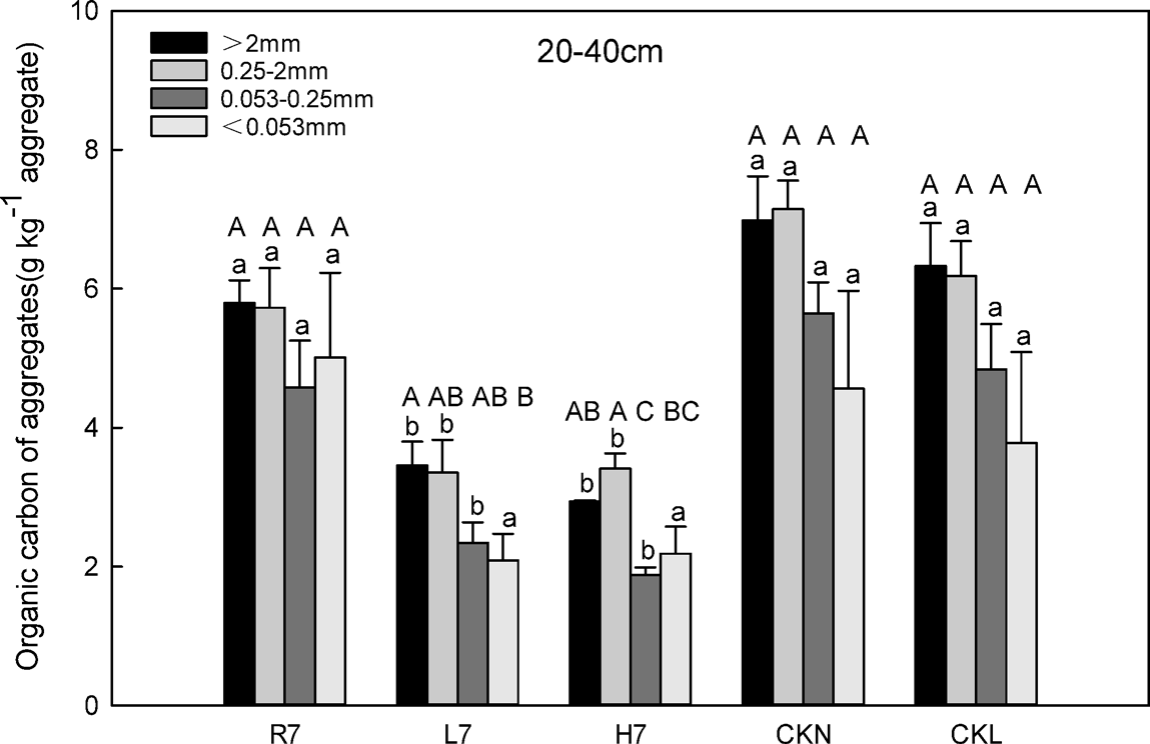
Soil aggregate-associated organic carbon characteristics (20–40 cm) under different land-use patterns. Error bars represent standard error (SE); lowercase letters indicate significant differences of the same particle size among different land-use patterns at *p* < 0.05 according to Duncan’s multiple range tests; capital letters indicate significant differences of the same land-use patterns among different particle sizes at *p* < 0.05 according to Duncan’s multiple range tests.

In the 20–40 cm layer, the organic carbon content of large macroaggregates, small macroaggregates, and microaggregates followed CKN > CKL > R7 > L7 > H7 (*p* < 0.05 for adjacent groups, Fig. 6), consistent with the surface layer. However, powder-cohesive agglomerates showed a reversed order: R7 > CKN > CKL > H7 > L7, with R7 significantly higher than CKL, H7, and L7 (*p* < 0.05). Among them, the organic carbon content of the large macroaggregates, small macroaggregates, and microagglomerates in reclaimed farmland was significantly higher than that of woodland and wasteland (*p* < 0.05), while there was no significant difference between woodland and wasteland. Additionally, the organic carbon content of the aggregates of each particle size in the 20–40 cm soil layer was lower than that of the 0–20 cm soil layer under every land-use pattern.

Different from the surface layer, the particle size distribution characteristics of the aggregate organic carbon in the 20–40 cm soil layer varied under different land-use patterns. The organic carbon content of the aggregates in reclaimed farmland, normal farmland, and normal woodland was approximately equal among the different particle sizes. Meanwhile, the organic carbon content of the coarse aggregates in reclaimed woodland was significantly higher than that of the silt-clay aggregates, and the organic carbon content of sM in the reclaimed wasteland was noticeably higher than that of the microaggregates.

#### Organic carbon contribution rates of soil aggregates

**Fig. 7:**
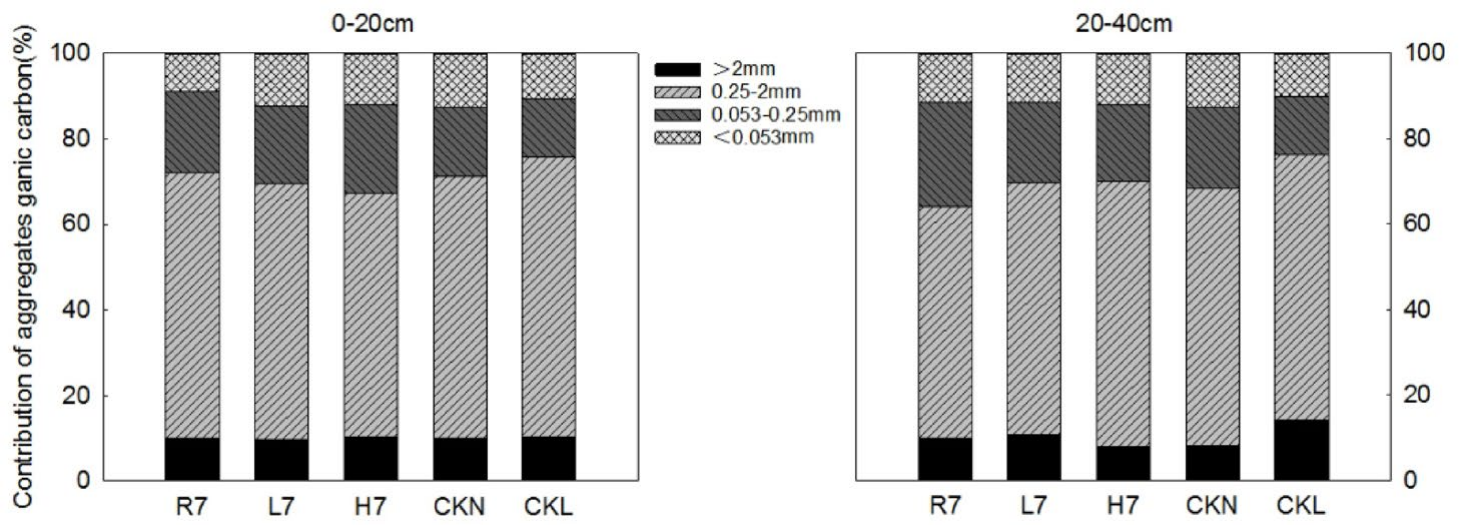
Soil aggregate-associated organic carbon contribution characteristics under different land-use patterns.

It can be seen from Fig. 7 that the relative order of the organic carbon contribution rates of the reclaimed soil aggregates of each particle size under the three land-use patterns was small: macroaggregates > microaggregates > silt-clay aggregates > large macroaggregates. The contribution rate of the fine-aggregate organic carbon was dominant (56.94–62.17% Fig. 7), which was completely consistent with the mass fraction characteristics of each particle size agglomerate, although their organic carbon contribution rates differed among the various land-use patterns. In the 0–20 cm soil layer, the relative order of the organic carbon contribution rates of the reclaimed soil aggregates (LM, sM) was R7 > L7 > H7. This was also due to the relative order of the mass fraction and organic carbon content of the reclaimed soil aggregates (LM, sM), which was farmland > woodland > wasteland after seven years of reclamation. Moreover, the contribution rate of the organic carbon of the reclaimed soil aggregates (LM, sM) of farmland (72.08%) after seven years of reclamation was slightly higher than that of the control farmland (71.09%), while the contribution rate of the organic carbon of the reclaimed soil aggregates (LM, sM) of the woodland (69.45%) after seven years of reclamation was still significantly lower than that of the control woodland (75.80%). Different from the surface layer, the relative order of the organic carbon contribution rates of the reclaimed soil aggregates (LM, sM) in the 20–40 cm soil layer was H7 > L7 > R7 among the different land-use patterns.

### Differences in organic carbon components of reclaimed soil aggregates under different land-use patterns

#### Characteristics of the free light fraction (free LF) organic carbon of soil aggregates

**Fig. 8:**
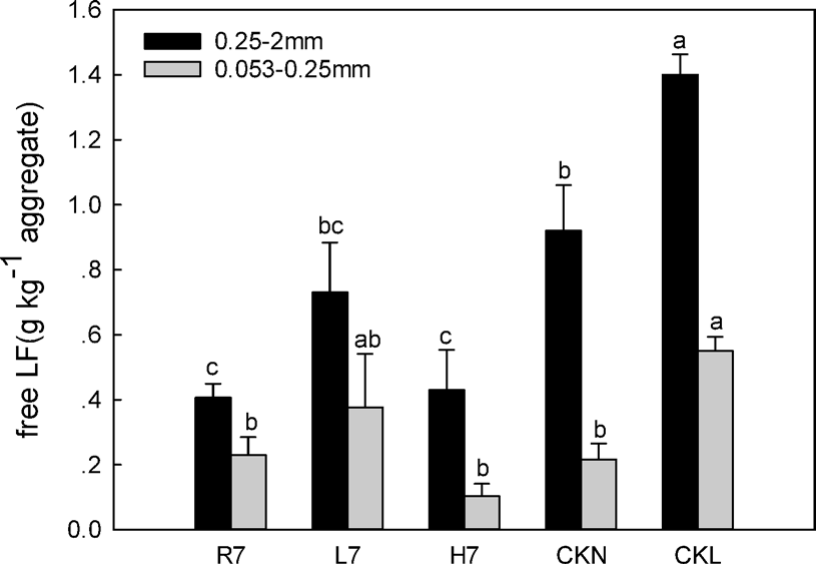
Free light fraction characteristics of soil aggregates under different land-use patterns. Error bars represent standard error (SE); lowercase letters indicate significant differences of the same component among different land-use patterns at *p* < 0.05 according to Duncan’s multiple range tests.

Different land-use patterns exhibited obvious effects on the free light fraction (free LF) organic carbon of soil aggregates, and their characteristics are presented in Fig. 8. CKL and CKN had significantly higher free LF carbon in sM than reclaimed sites (L7, H7, R7, *p* < 0.05), with the order CKL > CKN > L7 > H7 > R7 (Fig. 8). Woodland soils after seven years of reclamation showed a significantly higher fraction of free LF carbon (0.73 g kg⁻¹ aggregate) compared to farmland (0.41 g kg⁻¹ aggregate, *p* < 0.05), which clearly emphasizes the key contrast. This indicates that microbial activity likely dominated soil organic carbon (SOC) accumulation, as shown in Fig. 8. The fraction of free LF carbon in woodland soils was 1.78 times that of farmland and 1.70 times that of wasteland, further highlighting the difference in carbon storage capacity among these land-use types. Additionally, there was no large difference between the farmland and wasteland fractions. The free LF organic carbon contents of the sM under the three land-use patterns were all lower than that of the control farmland (0.92 g kg^−1^ aggregate) and woodland (1.40 g kg^−1^ aggregate). At the same time, the free LFs of the reclaimed soil and the control soil were noticeably higher than those of the farmland.

Among the different aggregate particle sizes, the free light fraction organic carbon content of the sM was higher than that of the micro aggregates under each land-use pattern. Nevertheless, the organic carbon content of the small soil macroaggregate-free LF of farmland after seven years of reclamation was 1.78 times that of the microaggregates, while it was 1.92 times lower than that of woodland and 4.3 times lower than that of wasteland.

#### Intra-aggregate particulate organic carbon (iPOM) characteristics

The coarse iPOM content of the small soil macroaggregates in farmland (2.49 g kg^−1^ aggregate) after seven years of reclamation was significantly higher than that of woodland (1.13 g kg^−1^ aggregate) and wasteland (1.06 g kg^−1^ aggregate), and was similar to that of the control farmland (2.61 g kg^−1^ aggregate) and control woodland (2.70 g kg^−1^ aggregate).

**Fig. 9:**
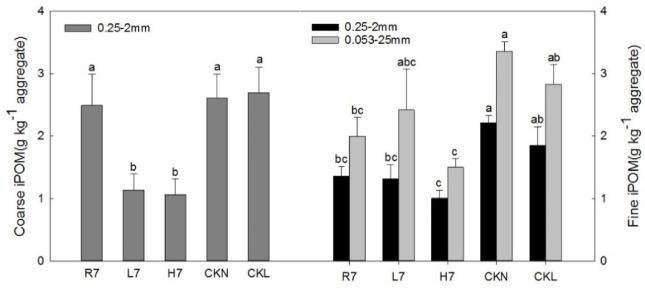
Soil intra-aggregate particulate organic carbon characteristics under different land-use patterns. Error bars represent standard error (SE); lowercase letters indicate significant differences of the same component among different land-use patterns at *p* < 0.05 according to Duncan’s multiple range tests.

The relative order of the fine iPOM content of the small macroaggregates (sM) was CKN > CKL > R7 > L7 > H7 in Fig. 9. This indicated that the fine iPOM concentration of sM in farmland after seven years of reclamation (1.36 g kg^−1^ aggregate) was larger than that of woodland (1.32 g kg^−1^ aggregate) and wasteland (1.00 g kg^−1^ aggregate), although both were significantly lower than the control farmland (2.21 g kg^−1^ aggregate) and control woodland (1.85 g kg^−1^ aggregate). The characteristics of the fine iPOM content of microaggregates under different land-use patterns were the same as those of the sM, although the content of fine iPOM (2.42 g kg^−1^ aggregate) in woodland soil microaggregates was higher than that in farmland (2.00 g kg^−1^ aggregate) after seven years of reclamation.

In terms of the aggregate particles, the fine iPOM content of the small soil macroaggregates under different land-use patterns was lower than that of microaggregates. However, the fine iPOM content of soil micro aggregates in woodland was 1.83 times that of the sM after seven years of reclamation, while it was 1.50 times higher than that of wasteland and 1.47 times higher than that of farmland.

#### Mineral-bound carbon (mineral-C) characteristics

**Fig. 10:**
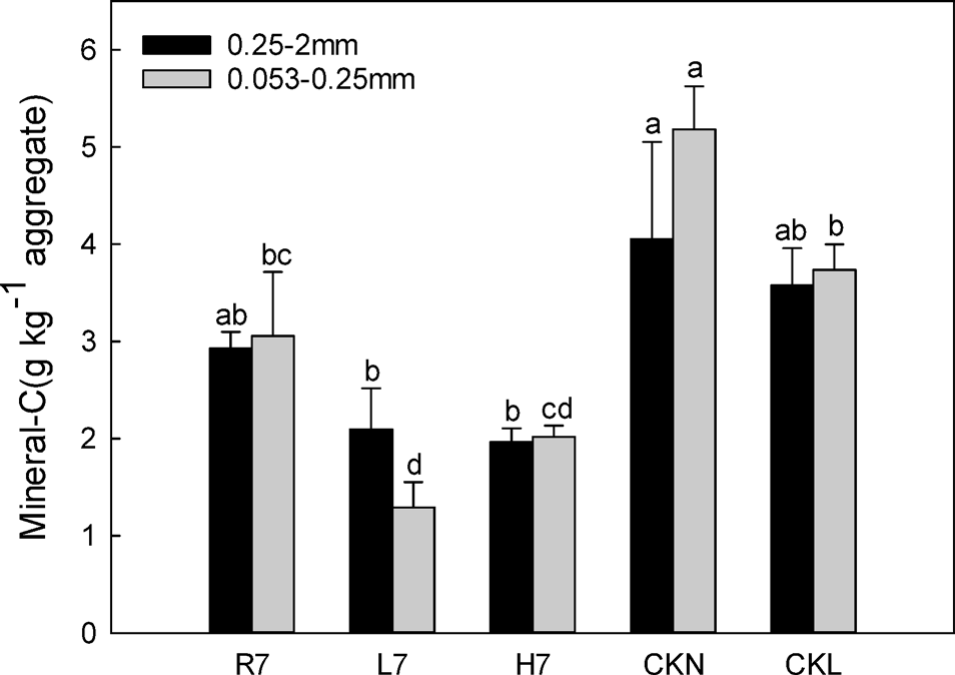
Soil aggregate mineral-associated organic carbon characteristics under different land-use patterns. Error bars represent standard error (SE); lowercase letters indicate significant differences of the same component among different land-use patterns at *p* < 0.05 according to Duncan’s multiple range tests.

The relative order of the mineral-bound carbon content of the small soil macroaggregates was CKN > CKL > R7 > L7 > H7 (*p* < 0.05) in Fig. 10. The mineral-C content (2.93 g kg^−1^ aggregate) of the sM in farmland was higher than that of woodland (2.10 g kg^−1^ aggregate) and wasteland (1.97 g kg^−1^ aggregate) after seven years of reclamation, while all three were lower than the contents of normal farmland and normal woodland. The features of the mineral-C content in soil micro aggregates under different land-use patterns were the same as those of sM, although the mineral-C content of soil micro aggregates of woodland (1.29 g kg^−1^ aggregate) was lower than that of wasteland (2.02 g kg^−1^ aggregate) after seven years of reclamation. In terms of aggregate particle size, except for woodland after seven years of reclamation, the mineral-C content of the sM under other land-use patterns was lower than that of the macro aggregates.

## Discussion

### Land use drives aggregate formation

Surface collapse can destroy soil aggregates during coal mining, while reclamation can promote the improvement of soil aggregate structure in mining areas^37^. Although the composition and quantity of aggregates showed subtle variations among land-use patterns, no significant differences were observed in their mass fractions, suggesting that land-use practices may have marginal influences rather than profound impacts on aggregate formation during the early reclamation stage. In this study, small macroaggregate (sM) mass fractions followed farmland (54.26%) > woodland (52.75%) > and wasteland (52.18%), though differences were not statistically significant. The content of sM in the reclaimed soil under the agricultural use pattern was the highest since the fertilizer input in the cultivation process increased the input of exogenous carbon and provided more cementing material for the formation of sM. Furthermore, legume rotation can promote soil agglomeration and increase the proportion of large water-stable aggregates, which is conducive to the formation and stability of aggregates. Woodland and wasteland lack the input of external organic carbon and mainly rely on the return of litter to increase soil nutrient content, as well as improve soil structure. Therefore, their sM mass fractions were lower than those of farmland. Owing to the difference in the amount of litter, the mass fraction of sM in forest soil was slightly higher than that of wasteland. In contrast to other reclamation studies (e.g., Stutler et al.^38^, which focused on long-term vegetation effects in reclaimed grasslands), our 7-year data showed subtle land-use impacts on aggregate characteristics (e.g., no significant differences in R0.25 or MWD among reclaimed land uses). This aligns with the conclusion that early-stage reclamation effects on aggregates are moderate, but vegetation development over time may drive more pronounced stability changes, as observed in longer-term research. Shrestha et al.^24^ Also illustrated that large soil aggregates increased significantly after 28 years of reclamation, and the restoration effect of reclamation in woodland was better than that of grassland. Nevertheless, Adeli’s^39^ research on the reclaimed soil in the Red Hills open-pit mining area in the United States revealed that the stability of soil aggregates in reclaimed grassland was higher than that of woodland, which is different from our results. This difference may be due to the different soil properties, climatic conditions, and vegetation types in different study areas. The soil, climatic, and vegetation conditions in Adeli’s study area may be more conducive for grassland vegetation to promote soil aggregate stability; whereas, in our study area, factors such as tillage fertilization of farmland and litter decomposition of woodland made farmland and woodland show different results in aggregate formation and stability than those in Adeli’s study. John 2004^40^ also pointed out that different land use modes can significantly affect the formation and stability of soil aggregates, which provides a theoretical basis for us to analyze the differences between the results of different studies. Compared with natural soils, natural forest soils rely on long-term bio-physical synergies (e.g., root secretions, earthworm activity) to form stable aggregates, such as Galindo et al.^41^ (2022) found that the percentage of biogenic aggregates in secondary forests was 42.7%, which was significantly higher than the exogenous carbon driving mechanism in the initial stage of reclaimed soils. In this study, the initial stage of reclaimed soil aggregate formation relied on anthropogenic interventions (e.g., fertilizer application, vegetation planting), whereas natural soils were structurally stabilized through long-term vegetation-microbe-mineral interactions (e.g., carbon accumulation in fungal detritus), so there were fundamental differences in the pathways of aggregate driving (short-term anthropogenic input vs. long-term ecological succession) and the mechanisms of carbon stabilization between the two. John 2004^40^ also pointed out that different land use modes can significantly affect the formation and stability of soil aggregates, which provides a theoretical basis for us to analyze the differences between the results of different studies. Therefore, the rational use of reclaimed soil in mining areas should be selected according to local conditions to be more conducive to the recovery and improvement of aggregates.

### Microbial vs. plant-mediated SOC pathways

After seven years of reclamation, the free light fraction (free LF) organic carbon content in woodland was significantly higher than that in farmland and wasteland (*p* < 0.05). In contrast, the organic carbon content of small macroaggregates and microaggregates was highest in farmland, followed by woodland and wasteland (Fig. 5), likely linked to agricultural management practices such as straw return. This suggests that the organic carbon in reclaimed woodland is in the early stage of rapid accumulation, with a large amount of litter covering the soil surface. During this stage, the accumulation rate of free LF organic carbon was faster than the total organic carbon accumulation. Moreover, the accumulation of free LF organic carbon in woodland soils is more likely driven by microbial activity, whereas in farmland, it is primarily influenced by surface vegetation residues^35^. This highlights the difference in carbon accumulation pathways between microbial and plant-driven processes in different land-use types. Qin et al.^42^. (2024) found that no-tillage affects organic carbon chemical composition by altering the source of carbon inputs in a non-mining system, which contrasts with the short-term carbon accumulation pattern of mining reclaimed soils in this study, suggesting that anthropogenic interventions (e.g., fertilizers, no-tillage) regulate carbon dynamics differently than natural restoration processes. In our study, the relative order of the soil aggregate free LF organic carbon of the reclaimed soil under different land-use patterns was R7 > L7 > H7, which may be due to the disturbance of coal mining and reclamation projects, the poor stability of soil aggregates, and the low organic carbon content. During the early stage of reclamation, it was the recovery stage that mainly improved the stability of soil aggregates and the accumulation of organic carbon. The destructive effect of farming on large aggregates was less than its accumulation. Thus, intra-aggregate particulate organic matter (iPOM) concentration in aggregates was primarily affected by large aggregate content and total organic carbon content after seven years of reclamation. Furthermore, the mineral-C characteristics in the agglomerates of the different land-use patterns were approximately the same as those of the iPOM concentration in the agglomerates, demonstrating that the large aggregate and total organic carbon content after seven years of reclamation mainly influenced the mineral-C content in the agglomerates. In addition, Adeli (2013) showed that the carbon content of reclaimed soils changes over time, with an increasing trend in total soil carbon content as the reclamation time increases. Despite the differences between the object and conditions of this study and the present study, both reflect the important effect of land use and reclamation measures on soil carbon content. This echoes the results of organic carbon accumulation and distribution in reclaimed soils under different land use practices in this study, further confirming the complexity of soil organic carbon accumulation and distribution as influenced by multiple factors. This suggests that microbial and plant-mediated pathways are intertwined in the process of soil organic carbon accumulation and that different land-use modes will lead to different roles of these two pathways in the dynamic changes of soil organic carbon.

### Limitations and future research

Although the present study reveals some of the patterns of soil aggregates and organic carbon under different land use modes after 7 years of reclamation, there are still some limitations. On the one hand, the period of this study is only 7 years after reclamation, which is not deep enough for the study of the long-term evolution of soil, and it is difficult to comprehensively grasp the dynamic changes of reclaimed soils on a longer time scale. On the other hand, the study area is relatively limited, and there are big differences in soil types, climatic conditions, and land use history in different areas, so the generalizability of the results of this study needs to be further verified. The development process of soil is essentially a coordinated evolutionary process of physical, chemical, and biological characteristics. In the future, we will further explore the aggregate process in reclaimed soil carbon fixation and the cooperative evolutionary characteristics of minerals, organic carbon, and microorganisms, revealing the nature of soil carbon fixation. On this basis, we may investigate methods for promoting this synergistic effect through reasonable utilization and effective measures to improve the reclaimed soil in mining areas. The results of this study emphasize the important role of soil aggregates in the fixation of organic carbon. Previous research has revealed that biochar not only has free particles in the soil but can also be located inside microaggregates and enriched in aggregates. On the one hand, the application of biochar contributes to the formation and stability of aggregates, while on the other hand, it benefits from the physical protection of aggregates to facilitate their long-term fixation^43,44^. Therefore, we could subsequently focus on the application of biochar in soil improvement, agricultural carbon sequestration, and emission reduction^45^.

### Implications for land reclamation strategies

The results of this study show that different land-use patterns significantly affect organic carbon components in soil aggregates (e.g., free light fraction carbon), while their impacts on aggregate distribution and stability remain subtle within the 7-year reclamation period. Based on this, in the formulation of land reclamation strategies, woodland reclamation should be emphasized. The advantages of woodland soil in terms of the organic carbon content in the fractions outside fine macro-aggregates, outside micro-aggregates, and in the total free light fraction imply its great potential in carbon sequestration, which can effectively enhance the ecological functions of reclaimed soil. In addition, considering the crucial role of soil aggregates in organic carbon fixation, biochar addition can be used as an effective improvement measure. Biochar can not only promote the formation and stability of aggregates but also achieve long-term retention through the physical protection of aggregates, thereby enhancing the soil’s carbon-sequestration capacity. Therefore, giving priority to woodland reclamation according to local conditions and reasonably adding biochar can help optimize land reclamation strategies and promote the ecological restoration and sustainable development of coal-mined subsidence areas.

## Conclusions

In this study, which focused on the impacts of different land-use patterns on reclaimed soil aggregates and organic carbon within a coal-mining subsidence area, the following findings were obtained:

In the 0–20 cm soil layer, based on the samples and analysis in this research, the relative order of the mass fractions of small soil macroaggregates under the three land-use patterns was farmland > woodland > wasteland. This indicates that farmland management practices (e.g., straw return) may have a relatively greater influence on the distribution of this specific aggregate size class, compared to woodland or wasteland under the conditions of this study. However, it is important to note that no statistically significant differences were found among land-use patterns for this metric. In addition, the organic carbon content of the reclaimed soil aggregates generally decreased with decreasing particle size. In this study, the contribution rate of organic carbon in small macroaggregates (sM) ranged from 56.94 to 62.17%, suggesting that sM represents a significant reservoir in the organic carbon contribution. However, the contributions of other factors and aggregate fractions cannot be completely ruled out.

The organic carbon content of the reclaimed soil surface aggregates under different land-use patterns was higher than that of the 20–40 cm soil layer. The investigation of the organic carbon content of the surface soil aggregates has significant implications in revealing the transformation and accumulation process of organic carbon in the reclaimed soil. However, as this study was based on a one-time sampling, long-term monitoring is essential to comprehensively characterize the dynamic variations in soil organic carbon content across seasons and years.

After seven years of reclamation, the free light fraction (free LF) organic carbon content in small soil macroaggregates, soil microaggregates, and total free LF in woodland was significantly higher than those of farmland and wasteland (*p* < 0.05). This observation is consistent with the hypothesis that microbial activity in woodland soil (potentially differing from farmland due to vegetation type and litter quality) may be more conducive to the accumulation of free LF organic carbon than management practices involving surface crop residues (e.g., straw return in farmland). However, this study did not directly measure microbial activity or community structure. The specific mechanisms driving these differences, likely involving variations in vegetation species, microbial community composition and function, and soil nutrient cycling dynamics, remain to be elucidated in future research.

To advance this field, future studies should integrate microbial community profiling and long-term monitoring to unravel the biotic drivers of aggregate-associated soil organic carbon (SOC) and validate the long-term benefits of woodland reclamation for SOC stabilization in mining soils. Prioritizing woodland reclamation may accelerate SOC sequestration, but sustained observation is critical to confirm these trends and inform adaptive management strategies.

## Data Availability

The data is available at Figshare: 10.6084/m9.figshare.29150993.v1.
